# The Serpin-like Loop Insertion of Ovalbumin Increases the Stability and Decreases the OVA 323–339 Epitope Processing Efficiency

**DOI:** 10.1021/acs.biochem.1c00095

**Published:** 2021-05-06

**Authors:** Daniel L. Moss, Ramgopal R. Mettu, Samuel J. Landry

**Affiliations:** † Department of Biochemistry and Molecular Biology, Tulane University School of Medicine, 1430 Tulane Avenue, New Orleans, Louisiana 70112, United States; ‡ Department of Computer Science, Tulane University, 6823 St Charles Avenue, New Orleans, Louisiana 70118, United States

## Abstract

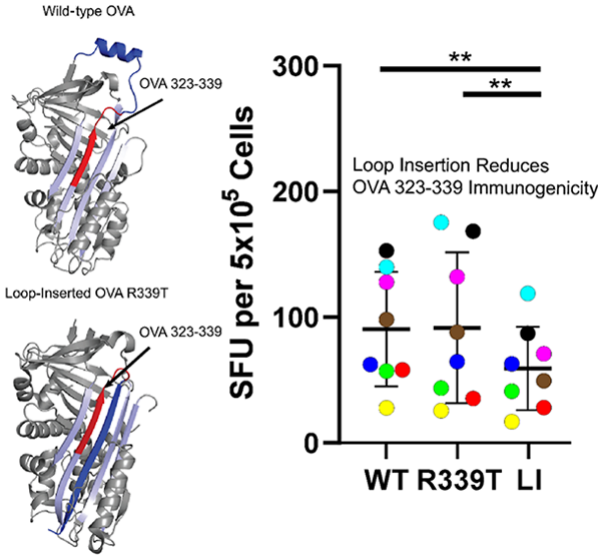

Chicken ovalbumin (cOVA) has been studied for decades primarily due to the robust genetic and molecular resources that are available for experimental investigations. cOVA is a member of the serpin superfamily of proteins that function as protease inhibitors, although cOVA does not exhibit this activity. As a serpin, cOVA possesses a protease-sensitive reactive center loop that lies adjacent to the OVA 323–339 CD4+ T-cell epitope. We took advantage of the previously described single-substitution variant, OVA R339T, which can undergo the dramatic structural transition observed in serpins, to study how changes in loop size and protein stability influence the processing and presentation of the OVA 323–339 epitope. We observed that the OVA R339T loop insertion increases the stability and protease resistance, resulting in the reduced presentation of the OVA 323–339 epitope *in vitro*. These findings have implications for the design of more effective vaccines for the treatment of infectious diseases and cancer as well as the development of more robust CD4+ T-cell epitope prediction tools.

Ovalbumin (OVA) is a member of the serpin family of proteins, which function as protease inhibitors, although OVA does not exhibit protease inhibitor activity. Serpins are described as metastable proteins because they are kinetically trapped in a conformation having a free energy higher than that of the conformation at the global free energy minimum.^1^ Serpins neutralize proteases by a remarkable structural transition, in which a highly flexible bait region, the reactive center loop (RCL), is preferentially cleaved; then the cleaved serpin assumes the more stable conformation by insertion of the newly released C-terminal portion of the loop into a large β sheet known as the A sheet. This loop insertion transition occurs too quickly for the enzyme to complete hydrolysis of the protease–serpin covalent intermediate, and the enzyme is subsequently inactivated by active-site distortion and disruption of the entire enzyme structure.^2^ Wild-type OVA does not exhibit protease inhibition activity or this dramatic structural change. However, substitution of arginine 339 with threonine allows loop insertion to occur without the appearance of protease inhibition activity.^3^ The RCL in OVA is located on the C-terminal flank of the OVA 323–339 peptide that contains the well-characterized OT-II epitope (Figure 1), providing an ideal protease cleavage site for the processing of this epitope. The loop insertion transition significantly reduces the amount of unstructured polypeptide on the C-terminal flank of the OVA 323–339 epitope (Figure 1). This alteration of the structural context of the OVA 323–339 peptide provides an ideal model system for studying how changing the structural context of an epitope affects its processing and presentation. Furthermore, our laboratory has developed a CD4+ T-cell epitope prediction tool that utilizes protein stability data to generate an antigen processing likelihood (APL) score that predicts CD4+ epitopes with significant accuracy.^4^ We hypothesized that loop insertion and shortening of the RCL decrease the processing efficiency of the OVA 323–339 peptide by increasing the overall protein stability and altering the local structural context of the OVA 323–339 peptide. Here we observed that loop insertion drastically stabilizes OVA R339T, increases its proteolytic resistance, and reduces the presentation of the OVA 323–339 epitope *in vitro*.

**Figure 1:**
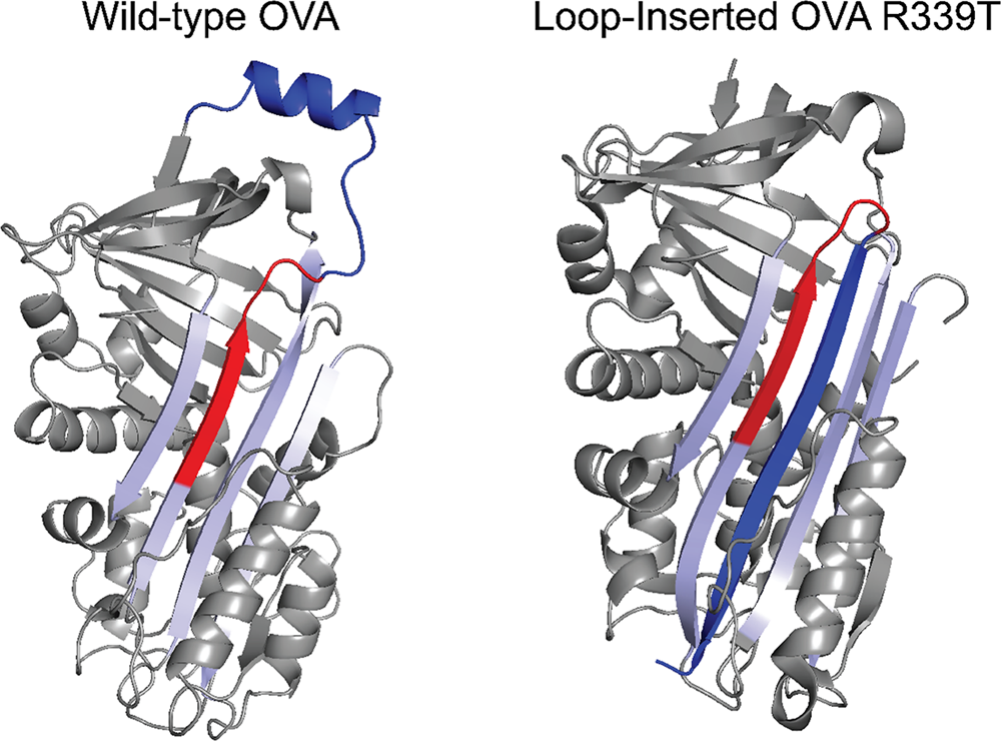
X-ray crystallographic structures of WT OVA (PDB entry 1OVA) and loop-inserted OVA R339T (PDB entry 1JTI). The reactive center loop (blue) is adjacent to the OVA 323–339 peptide (red). After proteolytic cleavage in the R339T variant, the reactive center loop inserts into the A sheet (light blue).

## Materials and Methods

### Cloning and Protein Purification

All recombinant ovalbumin variants used in this study were purified from *Escherichia coli* grown in autoinduction medium,^5^ with a typical yield of 10–15 mg/L of culture. Coding sequences for recombinant OVA variants were codon optimized for *E. coli*, synthesized, and cloned by Genscript using the NdeI and EcoRI sites of pET-22b(+) for lactose inducible expression and a C-terminal hexahistidine tag. Protein was purified from cell lysate as described previously for *Pseudomonas* exotoxin domain III, except growth was carried out at 37 °C.^6^ Briefly, cell pellets were resuspended in buffer A [50 mM Tris-HCl (pH 7.5) and 25 mM NaCl] and lysed with a French press at 16000 psi. The lysate was centrifuged at 38000*g* for 30 min and decanted. His-tagged OVA protein was purified by Ni-NTA chromatography and eluted in steps with buffer A and buffer B (A with 250 mM imidazole). Fractions containing His-tagged protein were pooled and further purified by anion-exchange chromatography and eluted with a gradient of buffer A and buffer C [50 mM Tris-HCl and 1 M NaCl (pH 7.5)]. Fractions containing OVA were pooled and concentrated by centrifugal filtration using a 10 kDa cutoff Amicon concentrator (Milipore-Sigma). OVA R339T LI was prepared as described previously.^3^ Briefly, the protein solution was adjusted to 1 mg/mL with 20 mM sodium phosphate buffer (pH 7.0) and incubated with 1 μg/mL porcine elastase (Fisher Scientific) for 4 h at room temperature. Proteolysis was stopped by the addition of 1 mM phenylmethanesufonyl fluoride (PMSF), and OVA R339T LI was purified by anion-exchange chromatography. Elastase cleavage under these conditions is highly specific for the P1–P1′ cleavage site within the RCL^3^ and exhibits >95% efficiency for generation of the OVA R339T LI as measured by sodium dodecyl sulfate–polyacrylamide gel electrophoresis (SDS–PAGE) and Coomassie staining. The protein concentration was calculated by absorbance at 280 nm using the extinction coefficient of ovalbumin (30590 M^–1^ cm^–1^).

### *In Vitro* Stability Analysis

For acid-induced unfolding experiments, the hydrophobic dye bis-ANS (4,4′-dianilino-1,1′-binaphthyl-5,5′-disulfonic acid, Invitrogen) was used to monitor protein unfolding by fluorescence spectroscopy with an excitation wavelength of 390 nm. Emission was scanned from 400 to 500 nm. Different pH conditions were generated using phosphate-citrate buffer, where 0.2 M dibasic sodium phosphate and 0.1 M citric acid were mixed until the desired pH was reached. Protein was mixed with dye in phosphate-citrate buffer ranging from pH 7.6 to 2.6 at concentrations of 1.0 μM protein and 10 μM dye in a 96-well plate format, and fluorescence data were collected using a Bio-Tek plate reader. Chemical denaturation experiments were performed in the same manner as acid denaturation experiments. Protein in phosphate-buffered saline (PBS) was mixed with guanidine-HCl in PBS in 0.25 M steps from 0 to 5 M and with 10 μM Bis-ANS in a 96-well plate format. Fluorescence data were analyzed as described previously to calculate the free energy of unfolding.^6−8^ Thermal denaturation experiments were performed with ovalbumin variants using a Malvern MicroCal VP-differential scanning calorimeter and a modified bis-ANS fluorescence assay. DSC scans began at 10 °C and ended at 90 °C at a scan rate of 90 °C/h with a 15 min prescan thermostat. The sample cell was filled with 7 μM protein in 20 mM sodium phosphate buffer (pH 6.0) for each scan. Data were analyzed with Malvern software and Microsoft Excel. Bis-ANS thermal denaturation was analyzed using the QuantStudio 6 Flex Real Time PCR system. The 96-well plates were filled with 100 μM bis-ANS dye and 10 μM OVA variant in 20 mM sodium phosphate buffer (pH 6.0). Thermal cycling began by chilling to 10 °C for 5 min and then scanning up to 90 °C at a rate of 1 °C/min. Raw fluorescence data were extracted into Microsoft Excel where they were trimmed and normalized. Normalized data were fit using Boltzmann sigmoidal nonlinear regression to calculate midpoints as *T*_m_ values.^9^

### Limited Proteolysis and Mass Spectrometry

Proteolysis reactions with cathepsin S were performed in phosphate-citrate buffer at pH 5.6 and 1 mM dithiothreitol. Each 20 μL reaction mixture contained 10 μg of protein and 0, 0.25, or 0.5 μg of recombinant human cathepsin S (Milipore-Sigma). Lysosomal extracts were prepared from RAW264.7 cells as described previously.^10^ Extract degradation reactions were performed in 50 mM sodium citrate (pH 5.9, 5.2, and 4.5) with 2 mM dithiothreitol. Each 200 μL reaction mixture contained 0.25 μg/μL protein and 0.4 μg/μL lysosomal extract. Reaction mixtures were incubated at 37 °C for the indicated time (30 min for cathepsin S), and reactions terminated by the addition of an equal volume of Bio-Rad Laemmli Sample Buffer containing 5 mM PMSF and 150 mM 2-mercaptoethanol. Samples were analyzed by SDS–PAGE using the Bio-Rad TGX gradient gel system and staining with Coomassie blue. Band intensities were analyzed with ImageJ and compared by two-way ANOVA and Tukey’s test for multiple comparisons. Mass spectrometry analysis of the gel slice was performed as described previously.^6^ Briefly, the chosen fragment was excised from the gel and destained using a 20 volume excess of 50 mM ammonium bicarbonate and 50% methanol for 20 min, twice. Destained gel slices were dehydrated by being incubated in a 20 volume excess of 75% acetonitrile for 20 min. Dried slices were then incubated in a 5 volume excess of 20 μg/mL mass spectrometry-grade trypsin dissolved in 50 mM ammonium bicarbonate at 37 °C overnight. Each sample was subjected to a 60 min chromatographic method employing a gradient from 2% to 25% acetonitrile in 0.1% formic acid (ACN/FA) over the course of 30 min, a gradient to 50% ACN/FA for an additional 10 min, a step to 90% ACN/FA for 8 min, and a re-equilibration into 2% ACN/FA. Chromatography was carried out in a trap-and-load format using a PicoChip source (New Objective, Woburn, MA); the trap column was a C18 PepMap 100, 5 μm, 100 Å column, and the separation column was a PicoChip REPROSIL-Pur C18-AQ, 3 μm, 120 Å, 105 mm column. The entire run was carried out at a flow rate of 0.3 μL/min. Survey scans were performed in the Orbitrap utilizing a resolution of 120000 between *m*/*z* 375 and 1600. Data-dependent MS2 scans were performed in the linear ion trap using a collision-induced dissociation (CID) of 25%. Raw data were searched using Proteome Discoverer 2.2 using SEQUEST specifying nonspecific cleavage to identify C-terminal peptides. The Protein FASTA database was SwissProt *Mus musculus* (TaxID = 10090) version 2017-07-05 with the OVA R339T sequence added for a total of 25098 sequences. Trypsin cleavage at lysine (K) and arginine (R) residues was specified as well as nonspecific cleavage to identify the C-terminal peptide. Static modifications included carbamidomethyl on cysteines (=57.021) and dynamic modification of oxidation of methionine (=15.9949). The parent ion tolerance was 10 ppm; the fragment mass tolerance was 0.02 Da, and the maximum number of missed cleavages was set to 2. Only high-scoring peptides (SEQUEST > 0.00) were considered utilizing a false discovery rate (FDR) of 1% calculated by Proteome Discoverer 2.2.

### IL-2 ELISpot

All animal experiments followed institutional guidelines and were approved by the Tulane Institutional Animal Care and Use Committee (IACUC). Animals were immunized by subcutaneous injection with 100 μg of OVA 323–339 peptide (Anaspec, Fremont, CA) dissolved in distilled water and emulfised 1:1 with complete Freund’s adjuvant. Fourteen days after immunization, mice were euthanized by CO_2_ asphyxiation, and spleens were aseptically removed and homogenized using gentleMACS C tubes (Miltenyi). IL-2 ELISpot was performed as described previously^6^ with minor modifications. Cells were plated at a density of 1 × 10^5^ or 5 × 10^5^ cells/mL in filter screen plates (Milipore-Sigma) and restimulated with the indicated concentration of OVA protein in complete RPMI medium (RPMI with 10% fetal bovine serum and 1% penicillin/streptomycin). The results are shown in spot forming units (SFU) per 5 × 10^5^ cells; significant responses were identified by the Wilcoxon signed-rank test, and mean SFU values for each condition were compared by repeated measures one-way ANOVA with Tukey’s test for multiple comparisons. For titration experiments, splenocytes were stimulated with a 5-fold dilution series starting at 60 μM protein and 20 μM peptide (final concentration in the 200 μL well). Spots at each antigen concentration were used to fit a nonlinear dose–response model, and log EC_50_ values were compared as shown in each figure.

### Conformational Stability Profile

Aggregate *z*-scores for residue-by-residue conformational stability were generated as described previously.^4^ Input data included *B*-factors, percent solvent accessible surface area, and COREX residue stabilities^11^ based on the structures of PDB entries 1OVA (WT OVA) and 1JTI (OVA R339T LI).

## Results

### Monitoring the Loop Insertion and Stability of Recombinant Ovalbumin Variants

**Figure 2:**
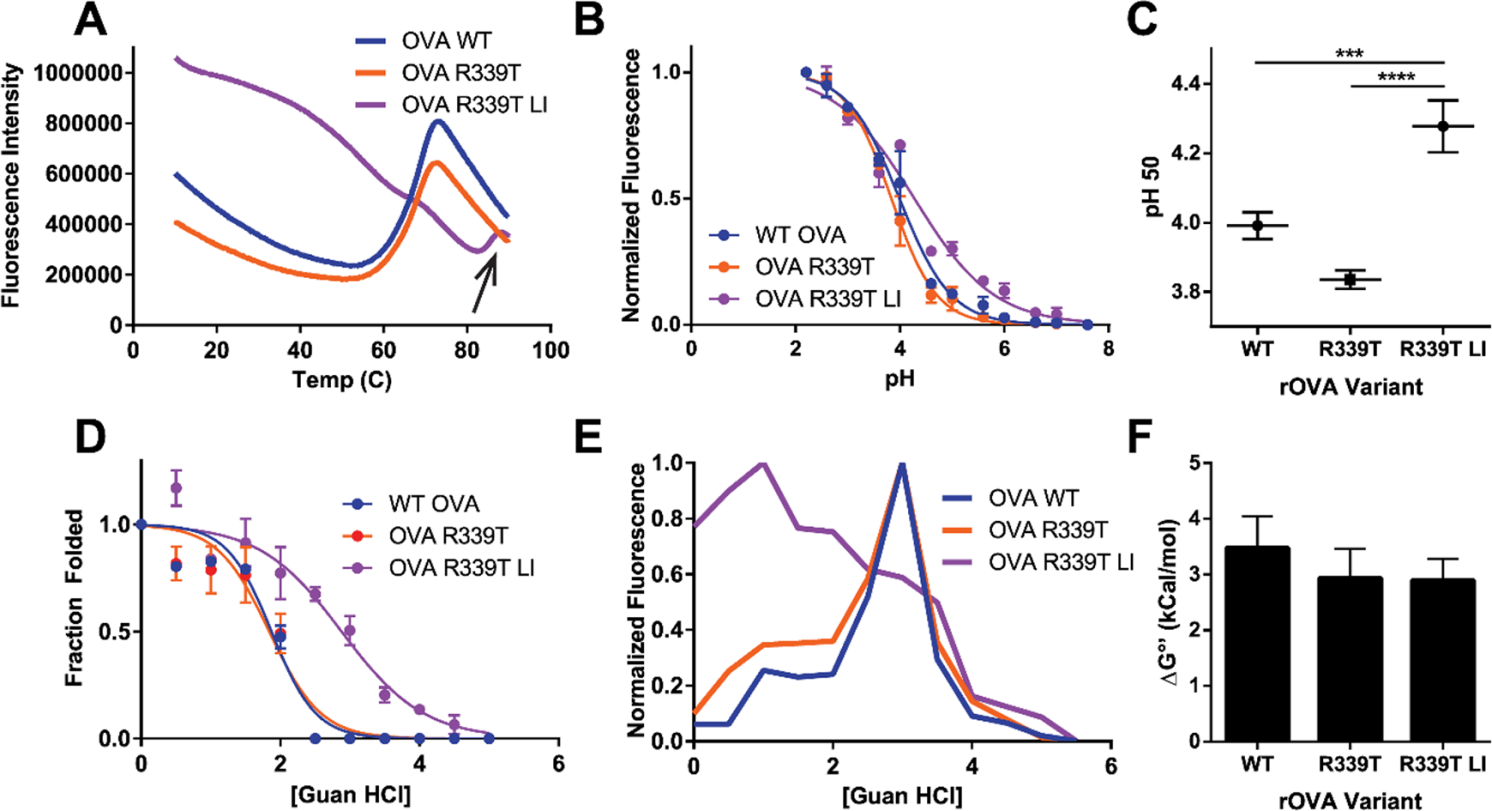
Loop insertion stabilizes OVA R339T against thermal denaturation but not acid- or denaturant-induced unfolding. (A) Temperature-induced unfolding curves for WT OVA, OVA R339T, and loop-inserted OVA R339T monitored by fluorescent dye binding, representative of at least three independent experiments. The arrow indicates the unfolding transition of OVA R339T LI. (B) Acid-induced unfolding curves for OVA variants (*n* = 3). (C) Analysis of best-fit pH_50_ values. Error bars indicate the standard error; asterisks indicate significance by one-way ANOVA (***p < 0.001; ****p < 0.0001). (D) Chemical denaturation of OVA variants using guanidine hydrochloride [Guan HCl] and reported by bis-ANS fluorescence (*n* = 3). The ratio of the intensity at a given [Guan HCl] to that at zero [Guan HCl] was plotted for each concentration, and the data were fit to solve for the free energy of unfolding at zero [Guan HCl] (Δ*G*°′). (E) Normalized bis-ANS fluorescence of OVA variants as a function of guanidine concentration. Loop-inserted OVA R339T exhibits decreasing fluorescence as the guanidine concentration increases. (F) Analysis of Δ*G*°′ values determined in panel D. Error bars indicate the standard error (*n* = 3).

For our studies, we utilized recombinant wild-type (WT) and OVA R339T variants produced in *E. coli*. The structure of loop-inserted P1–P1′ cleaved ovalbumin has been described previously and is prepared using specific protease treatment conditions.^3^ Briefly, purified recombinant OVA R339T is treated with elastase at pH 6.0 for 4 h at room temperature before the reaction is stopped by the addition of phenylmethanesulfonyl fluoride, and the protease is separated from the now P1–P1′ cut, loop-inserted OVA R339T (OVA R339T LI) by ion-exchange chromatography. SDS–PAGE analysis of this reaction shows that >95% of OVA R339T is cleaved and converted to the loop-inserted conformation (data not shown). The P1–P1′ elastase cut site is located between alanine 353 and serine 354.^12^ The loop insertion transition can be monitored by the increase in midpoint temperature for thermal denaturation via differential scanning calorimetry (DSC) or a fluorescent bis-ANS dye-based assay.^9^ We prepared OVA R339T LI by the published method and used both DSC and the fluorescence-based assay to confirm loop insertion by thermal denaturation (Figure 2A, Table 1, and Figure S1). Our results closely match those previously reported, confirming that the loop-inserted conformation of OVA R339T had been achieved. The loop-inserted thermal unfolding transition occurred above 80 °C and has a small enthalpy compared to those of the WT and R339T variants. Next we tested the hypothesis that loop insertion stabilizes OVA against acid- and denaturant-induced unfolding using the bis-ANS fluorescence assay we have employed previously with other antigens.^6^ We observed that OVA R339T LI unfolded at a pH slightly higher than those of both WT and OVA R339T (Figure 2B,C). However, all three proteins unfolded at a pH below the late endosomal pH of 4.5,^13^ suggesting that they can maintain a mostly native or native-like conformation throughout the endosomal system and within the antigen-processing compartment. Denaturant-induced unfolding yielded a broader unfolding curve (Figure 2D) and revealed that OVA R339T LI bound the most dye at a low denaturant concentration (Figure 2E). Using these unfolding data, we calculated and observed no difference in the Gibbs free energy of unfolding among WT, OVA R339T, and OVA R339T LI (Figure 2F).

**Table 1:** Midpoint Thermal Denaturation (*T*_M_) Values for the Indicated OVA Variants Determined by the Fluorescence Assay and Differential Scanning Calorimetry in Degrees Celsius

	this study (bis-ANS; *n* = 8)	this study (DSC; *n* = 3)	ref (3)
OVA WT	67.9	74.9	72.4
OVA R339T	67.2	ND	72.4
OVA R339T LI	85.6	89.5	88.2

### Characterizing the Proteolytic Susceptibility of Ovalbumin Variants

**Figure 3:**
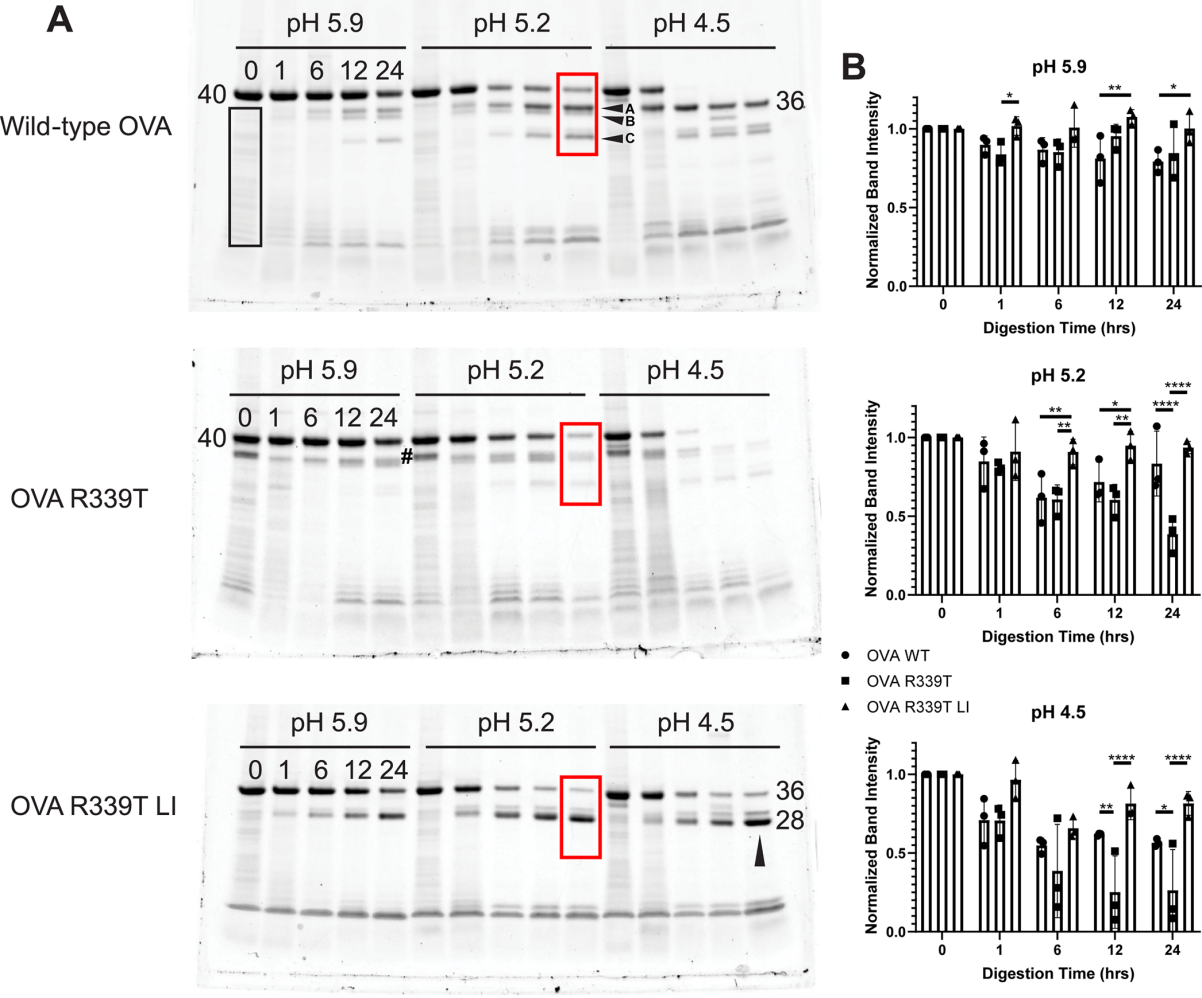
Limited proteolysis of OVA variants by lysosomal extracts. (A) Proteolysis reactions were sampled at the indicated time points (hours) before analysis by SDS–PAGE and Coomassie staining. A major fragment of loop-inserted OVA R339T exhibits significant resistance to further degradation compared to that of intact WT or OVA R339T or fragments thereof. Arrowheads indicate fragments that were selected for identification by trypsin digestion and mass spectrometry. Numbers adjacent to fragments indicate molecular weights in kilodaltons determined from regression analysis of markers in Figure 4. The band indicated by # is a full-length disulfide-bonded species that was resistant to a reducing agent at low pH and was included in quantification of intact protein. (B) Intensities for OVA, OVA R339T, or OVA R339T LI were normalized to the 0 h time point and then compared by two-way ANOVA and Tukey’s test for multiple comparisons; error bars indicate the standard deviation (*n* = 3). A single asterisk indicates a *p* value of <0.05. Two asterisks indicate a *p* value of <0.01. Three asterisks indicate a *p* value of <0.001. Four asterisks indicate a *p* value of <0.0001. The black box indicates proteins from the RAW264.7 lysosomal extract; red boxes mark bands that were quantified for analysis in panel B.

**Figure 4:**
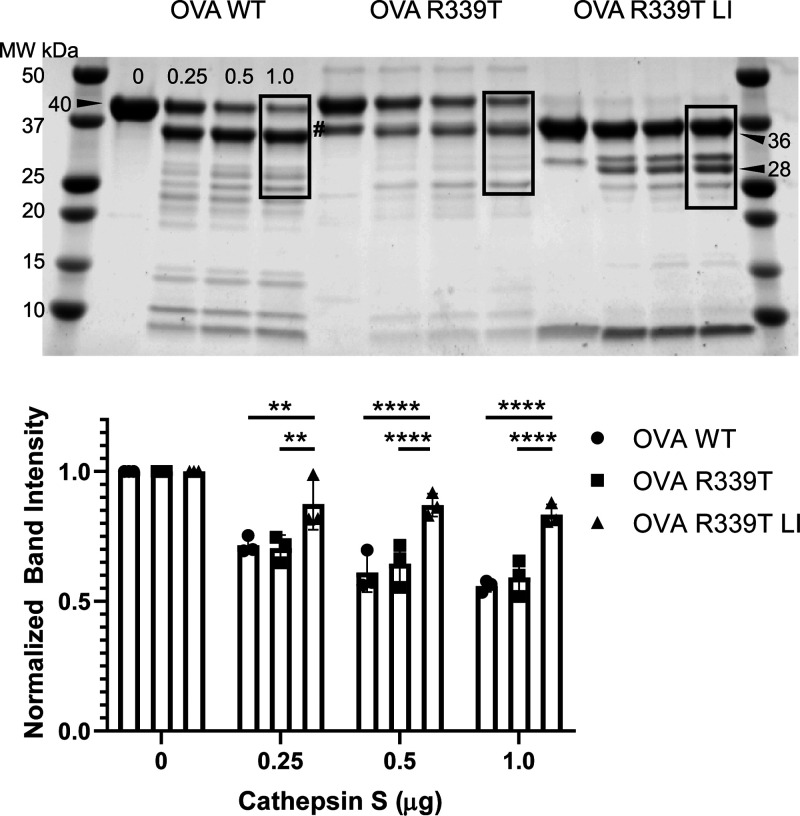
Limited proteolysis of OVA variants by cathepsin S at pH 5.6. OVA variants were incubated with the indicated amount of cathepsin S for 30 min at 37 °C before analysis by SDS–PAGE and Coomassie staining. Numbers indicate calculated molecular weights from gel analysis software. Intensities for boxed bands were quantified, normalized to the lane with zero protease, and plotted below the gel. Intensities were compared by two-way ANOVA and Tukey’s test for multiple comparisons; error bars indicate the standard deviation (*n* = 3). Asterisks indicate a *p* value of <0.05. The band indicated by # is a full-length disulfide-bonded species that was resistant to the reducing agent at low pH and was included in quantification of intact protein.

Our hypothesis of antigen processing holds that flexible, unstable portions of antigens are sites for proteolytic cleavage and that adjacent stable segments will be presented to CD4+ T cells. Previous work by our laboratory and others has shown that conformational stability has an impact on protein immunogenicity, with examples of antigens or antigen domains in which conformational stability enhanced immunogenicity^6,14,15^ and others in which conformational stability decreased immunogenicity.^7,13,16^ In the case of OVA, we hypothesized that the stabilized OVA R339T LI would exhibit an overall increase in proteolytic resistance compared to that of native OVA R339T and WT OVA. To test this, we performed limited proteolysis experiments with lysosomal extracts from a mouse macrophage cell line RAW264.7 (Figure 3) and with the lysosomal protease cathepsin S (Figure 4).

**Figure 5:**
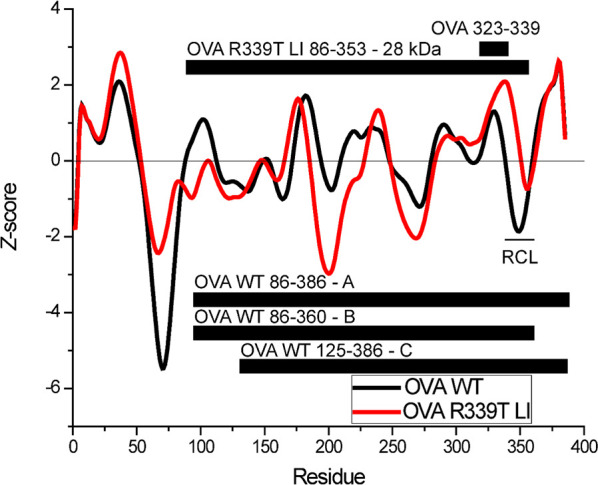
Conformational stability profiles for WT (black) and loop-inserted OVA R339T (red). Aggregate *z* scores are plotted for each protein. The small black bar indicates the location of the OVA 323–339 peptide, and the RCL is indicated. Large black bars indicate OVA fragments indicated in Figure 3 and identified by trypsin digestion and mass spectrometry.

Proteolysis of ovalbumin variants by lysosomal extracts was conducted over a time course of 24 h at three different pH environments corresponding to early (pH 5.9), middle (pH 5.2), and late (pH 4.5) endosomal environments^13^ (Figure 3A). Proteolytic fragments derived from WT OVA persisted longer than those produced from OVA R339T but not as long as a 28 kDa fragment (Figure 3, arrow) derived from OVA R339T LI. This fragment was excised from the gel and subjected to trypsin digestion followed by mass spectrometry to determine the approximate cleavage site (Table S1). Tryptic OVA R339T LI-derived peptides aligned with the core of the protein, spanning residues 86–353 and containing the OVA 323–339 peptide (Figure 5 and Figure S2). Residue 353, alanine, is the P1 residue that comprises the P1–P1′ elastase site used to generate OVA R339T LI. A similar set of persisting fragments were observed to be generated from WT OVA, fragments A–C (Figure 3, box and arrows). These were also analyzed by trypsin digestion and mass spectrometry (Table S2). Tryptic peptides from fragment A spanned the entirety of the C-terminal portion of the molecule resulting from an approximate cleavage site near residue 86 (Figure 5). Peptides from fragment B spanned the C-terminal portion of the molecule from residue 86 to 360, suggesting that this fragment may be derived from fragment A after cleavage within the RCL region, although not at the same P1–P1′ elastase site, as this site was included in a tryptic peptide (Figure 5 and Table S2). Fragment C from WT OVA contained tryptic peptides extending from approximately residue 125 to the C-terminus (Figure 5) and therefore has not experienced cleavage within the RCL.

For analysis of destructive proteolysis, band intensities for all large OVA-derived fragments that contain the OVA 323–339 peptide were quantified (Figures 3 and 4, boxes). Following treatment of OVA proteins with lysosomal extracts at pH 5.9, major fragments from OVA R339T LI accumulated more than those from WT OVA at 1, 12, and 24 h (Figure 4B). At pH 5.2, fragments of OVA R339T LI were more abundant than those from either WT or OVA R339T after incubation for 6 and 12 h. After 24 h, fragments from WT and OVA R339T LI were more abundant than OVA R339T-dervied fragments, while no difference was observed between WT and OVA R339T LI. At pH 4.5, WT- and OVA R339T LI-derived fragments were more abundant than OVA R339T fragments after 12 and 24 h, while no difference between WT and OVA R339T LI was observed.

Various proteolytic activities in the complex lysosomal extracts may be differentially affected by pH, potentially confounding an interpretation based on antigen conformation. Thus, we performed a similar analysis of variant susceptibility to proteolysis by the single protease, cathepsin S, a well-documented antigen-processing protease. Limited proteolysis with cathepsin S at pH 5.6 revealed that OVA 323–339-containing polypeptides of OVA R339T LI were more resistant to fragmentation than those of WT or OVA R339T (Figure 4).

### Analysis of Antigen Processing Likelihood

We have previously reported an epitope prediction algorithm that combines multiple types of protein conformational stability data for the generation of a residue-by-residue antigen processing likelihood score.^4^ Stability data were collected using crystal structure 1OVA of chicken ovalbumin^21^ and using crystal structure 1JTI of OVA R339T LI.^3^ Aggregate conformational stability results are shown in Figure 5 and displayed as individual residue *z* scores. For WT OVA, the OVA 323–339 peptide is contained in a stable region characterized by an increased stability, N-terminally adjacent to the reactive center loop, which is evident as the large negative dip in the aggregate stability *z* score (Figure 5). For OVA R339T LI, the OVA 323–339 region is significantly stabilized, compared to that for WT OVA, and the now-inserted RCL is much shorter and less flexible (11 residues of negative aggregate *z* score). We also note that the N-terminal cleavage site associated with the generation of the OVA R339T LI 28 kDa fragment, and both WT OVA fragments A and B, coincides with a large flexible segment. Additional flexible segments, especially in OVA R339T LI, are expected to offer potential protease nick sites, but the much smaller fragments resulting from such cleavages may not be sufficiently stable to accumulate as well as the larger fragments.

### Processing of OVA 323–339 from Ovalbumin Variants

**Figure 6:**
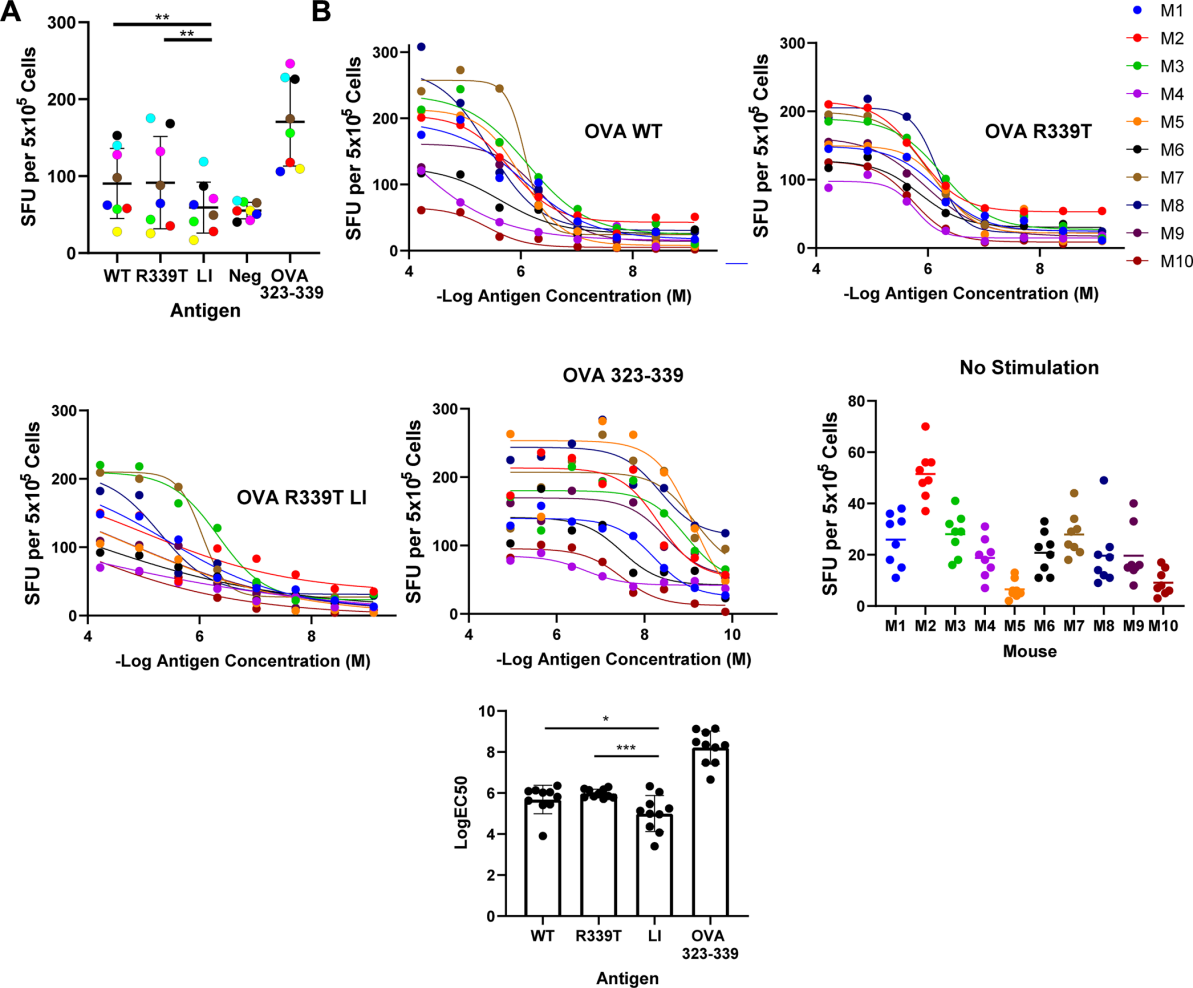
Reduced *in vitro* processing and presentation efficiency of the OVA 323–339 peptide from loop-inserted OVA R339T. (A) Splenocytes from eight BALB/c mice immunized with the OVA 323–339 peptide emulsified in complete Freund’s adjuvant were provided with the indicated OVA variant, and IL-2 production was measured by ELISpot. Asterisks indicate significance by repeated-measures one-way ANOVA (*p < 0.05; ***p < 0.001). Solid lines indicate means, and error bars indicate the standard deviation. (B) Splenocytes from 10 BALB/c mice immunized with the OVA 323–339 peptide emulsified in complete Freund’s adjuvant were provided with the indicated OVA variant in a 5-fold dilution series starting at 60 μM, and IL-2 production was measured by ELISpot. Spots at each concentration were fit to a nonlinear dose–response model, and log EC_50_ values were compared. Asterisks indicate significance by repeated measures one-way ANOVA (*p < 0.05; ***p < 0.001), and error bars indicate the standard deviation.

A class II restricted T-cell epitope corresponding to OVA 323–339 has been reported in multiple epitope mapping studies undertaken with several strains of inbred mice. In all cases, the mice were immunized with intact OVA emulsified in complete Freund’s adjuvant (CFA).^17−20^ For this analysis of OVA processing, we utilized BALB/c mice immunized with the OVA 323–339 peptide emulsified in CFA and monitored IL-2 ELISpot formation upon restimulation with OVA proteins (Figure 6). In one experiment, splenocytes from eight immunized mice were restimulated with WT OVA or variant at a concentration of 2.5 μM or OVA 323–339 peptide at 1 μM. Spot counts in response to no stimulation were subtracted from those produced in response to OVA stimulation. IL-2 spots produced in response to each OVA variant were counted and compared by repeated measures ANOVA. We observed no difference in IL-2 spot formation when WT or OVA R339T was provided; however, a significant reduction in the level of spot formation was observed when splenocytes were stimulated with OVA R339T LI (Figure 6A). In a second experiment, the amount of protein or OVA 323–339 peptide was titrated in a 5-fold dilution series starting at 60 μM protein and 20 μM peptide, and CD4+ T-cell responses were again measured by IL-2 ELISpot. IL-2 spots were counted for each dilution and each animal (*n* = 10) and used to fit a nonlinear dose–response model. Comparison of log EC_50_ values revealed a significant reduction in response when cells were stimulated with OVA R339T LI, compared to stimulation with either WT OVA or OVA R339T (Figure 6B). Restimulation with either WT OVA or OVA R339T generated a mean log EC_50_ value of 5.9, whereas restimulation with LI OVA R339T generated a mean log EC_50_ value of 5.0. These results suggest that loop insertion and stabilization reduce the processing and presentation efficiency of the OVA 323–339 peptide from the OVA protein.

## Discussion

In this study, we tested the hypothesis that loop insertion by the OVA R339T variant reduces the processing efficiency of the OVA 323–339 peptide. We were able to replicate a previous observation that loop insertion in OVA R339T significantly stabilizes the protein against thermal denaturation. Interestingly, OVA R339T LI exhibited broader temperature, acid, and denaturant-induced unfolding curves and bound high levels of bis-ANS dye at low temperatures and low denaturant concentrations (Figure 2A,E), while the WT and R339T OVA proteins exhibited traditional dye binding characteristics, suggesting that the loop-inserted conformation of the protein exists as a molten globule.^22−25^ Molten globules typically exhibit greater disorder than corresponding native states, but some molten globules are associated with increased conformational stability and significant protease resistance and have even been crystallized.^26,27^ We also observed that loop insertion drastically increases resistance to antigen-processing proteases. Limited proteolysis of OVA R339T LI with RAW lysosomal extracts or cathepsin S revealed the accumulation of an approximately 28 kDa proteolytic fragment that was less prominent in digests of WT and the R339T OVA variant (Figures 3 and 4). Using trypsin digestion and mass spectrometry, we identified the protease-resistant OVA R339T LI fragment as spanning residues 86–353 (Table S1 and Figure S2), which contains the OVA 323–339 epitope. We also observed the accumulation of other large OVA-derived proteolysis fragments generated from WT OVA. These were also identified by trypsin digestion and mass spectrometry (Table S2) and found to contain the OVA 323–339 peptide. For comparison of the remaining protein following limited proteolysis, band intensities for these various OVA 323–339 peptide-containing fragments were summed together with that of intact proteins. OVA R339T LI and relevant fragments were significantly more resistant to cathepsin S proteolysis, compared to those of either WT or OVA R339T (Figure 4). However, when subjected to proteolysis by the lysosomal extract, major differences between OVA R339T LI and WT or OVA R339T emerged only at pH 5.2 (Figure 3). This may be significant for the immunogenicity of the OVA 323–339 peptide because the MHCII contents in early and late endosomal compartments (pH 5.9 and 4.5, respectively) are low, compared to the intermediate endosome stage (pH 5.2).^13,28^ Taken together, these observations suggest that loop insertion stabilizes OVA R339T and proteolytic fragments that contain the OVA 323–339 peptide at the stage of endosomal processing where optimal peptide loading of MHCII is believed to occur.

Loop insertion in OVA R339T reduced the processing and presentation of the OVA 323–339 epitope. To focus on the OVA 323–339 peptide, BALB/c mice were primed with the OVA 323–339 peptide, and then the resulting splenocytes were restimulated with recombinant OVA proteins *in vitro* (Figure 6). A reduction in restimulation efficiency was observed with OVA R339T LI, compared to those of intact OVA R339T or WT OVA. These results suggest that the OVA 323–339 peptide is less efficiently processed and presented from the stabilized OVA R339T LI.

After loop insertion, residues contained within the reactive center loop are inserted into the A sheet, leaving a much shorter stretch of residues connecting to the β strand containing the OVA 323–339 peptide residues (Figure 1). Prior to loop insertion, the RCL is a preferred protease cleavage site due to its length and unstructured nature in serpins. In OVA, the RCL has some secondary structure, but it is still cleaved by lysosomal extracts (Figures 3 and 5). Preferential cleavage by elastase is necessary to generate the loop-inserted variant of OVA R339T, further indicating the preference of proteases for the RCL. A mixture of lysosomal proteases also exhibited a preference for an N-terminal cleavage site around residues 75–85, demonstrated by the accumulation of a fragment A spanning from this site to the C-terminus of WT OVA (Figures 3 and 5). This N-terminal site may be preferred over the RCL for initial proteolysis because the resulting fragment accumulated more than any fragments resulting from cleavage within the RCL (Figures 3 and 5).

In the case of OVA R339T LI, we speculate that loop insertion significantly reduces or even completely blocks further proteolysis at the RCL, therefore preventing release of the OVA 323–339 peptide. Previous studies of limited proteolysis suggest that proteolytic nicking by typical proteases, including cathepsin S, requires a flexible loop of 12–15 amino acid residues.^29^ Thus, the RCL in OVA R339T LI is no longer a preferred site for proteolytic antigen processing for the presentation of the OVA 323–339 peptide. The shrinkage of a flexible loop resulting in protease resistance has been described for other antigens such as *Pseudomonas* exotoxin A and T4 bacteriophage Hsp10.^6,30,31^ The obstruction of proteolytic processing combined with conformational stabilization may prevent extraction of this peptide for loading onto MHCII and presentation to CD4+ T cells. This result is reminiscent of the reduced immunogenicity in stabilized variants of the birch pollen allergen Bet v 1 due to inefficient processing of T-cell epitopes.^13^

In summary, proteolytic cleavage and insertion of the reactive center loop in OVA R339T result in a large increase in global OVA stability as well as loop shortening adjacent to the OVA 323–339 peptide. This change in stability and local epitope structural context reduces the processing efficiency of the OVA 323–339 peptide *in vitro* and may reduce CD4+ T-cell priming efficiency *in vivo*. Future studies will explore how loop size and conformation affect immunogenicity and examine implications for the design of more accurate CD4+ T-cell epitope prediction tools.

## Supporting Information Available

Figures S1 and S2 and peptides identified by mass spectrometry (Tables S1 and S2) (PDF)

## Notes

The mass spectrometry proteomics data reported here have been deposited to the ProteomeXchange Consortium via the PRIDE^32^ partner repository with the data set identifier PXD021756 and DOI: 10.6019/PXD021756.

## Abbreviations

MHCII: MHC class II molecule
GdnHCl: guanidine hydrochloride
PMSF: phenylmethanesulfonyl fluoride
PDB: Protein Data Bank
ANOVA: analysis of variance
APL: antigen processing likelihood
OVA: ovalbumin
cOVA: chicken ovalbumin
OVA R339T LI: loop-inserted OVA R339T
RCL: reactive center loop
bis-ANS: 4,4′-dianilino-1,1′-binaphthyl-5,5′-disulfonic acid.
