# Supplementary material for: The Serpin-like Loop Insertion of Ovalbumin Increases the Stability and Decreases the OVA 323–339 Epitope Processing Efficiency
Source: Biochemistry. 2021 May 6;60(20):1578–86. doi: 10.1021/acs.biochem.1c00095 (PMC8253479; doi:10.1021/acs.biochem.1c00095)
Supplement: Supplementary file 1 — bi1c00095_si_001.pdf [file bi1c00095_si_001.pdf]

Supporting Information

The Serpin-like Loop-insertion of Ovalbumin Increases Stability and Reduces OVA 323-339 Epitope Processing Efficiency

Daniel L. Moss[1], Ramgopal R. Mettu[2], Samuel J. Landry[1*]

1. Department of Biochemistry and Molecular Biology, Tulane University School of Medicine, 1430 Tulane Avenue, New Orleans, LA 70112, USA
2. Department of Computer Science, Tulane University, 6823 St Charles Avenue, New Orleans, LA 70118, USA

*Correspondence:

Samuel J. Landry

landry@tulane.edu

Contents:

Figure S1. Temperature induced unfolding of OVA WT and R339T LI measured by differential scanning calorimetry.

Figure S2. Crystal structure of loop inserted OVA R339T (PDB 1JTI) colored to show the approximately 28 kDa fragment (blue) identified by trypsin digestion and mass spectrometry in Figure 4.

Table S1. OVA R339T peptides identified by mass spectrometry after trypsin digestion of the 28-kDa proteolytic fragment identified in Fig. 4.

Table S2. WT OVA peptides identified by mass spectrometry after trypsin digestion of band A (top) band B (middle) and band C (bottom) proteolytic fragments identified in Fig. 4

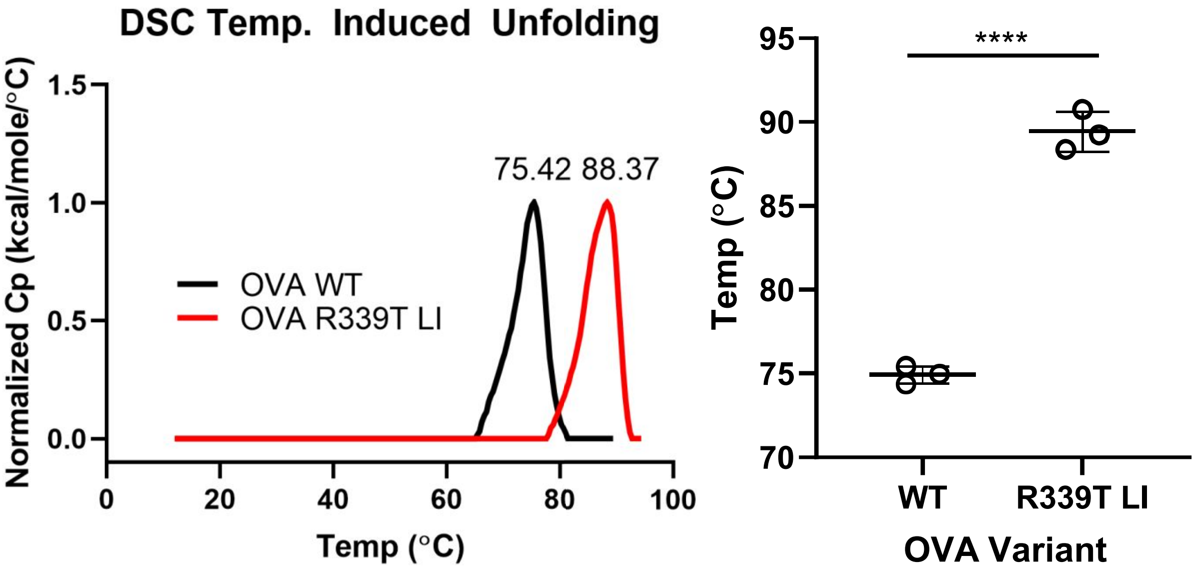

Figure S1. Temperature induced unfolding of OVA WT and R339T LI measured by differential scanning calorimetry. Representative unfolding curves graphed as a function of temperature. Scans were performed in triplicate and melting temperature values were compared by two-tailed t-test. Mean Tm values are shown, error bars indicate standard deviation, asterisks indicate p valve < 0.0001.

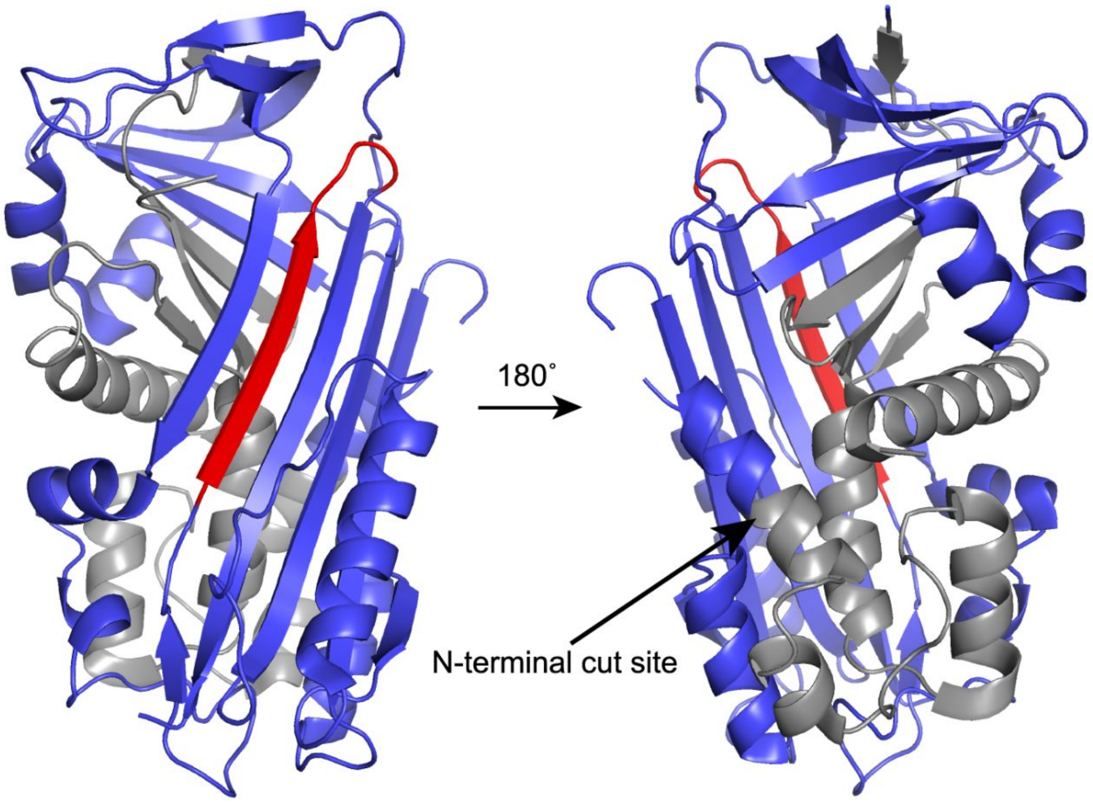

Figure S2. Crystal structure of loop inserted OVA R339T (PDB 1JTI) colored to show the approximately 28 kDa fragment (blue) identified by trypsin digestion and mass spectrometry in Figure 4. The red portion indicates the OVA 323-339 peptide. Approximate N-terminal cleavage site is indicated by arrow.

Table S1. OVA R339T peptides identified by mass spectrometry after trypsin digestion of the 28-kDa proteolytic fragment identified in Fig. 4. Tryptic peptides that overlap with OVA 323-339 are highlighted.

Annotated Sequence	Modifications	# PSMs	Delta m/z [Da]	Charge	m/z [Da]	XCorr	Positions
[H].AAHAEINEAGTEVVGSAEAGVDAA.[S]		46	0.00042	2	1120.02771	6.39	[330-353]
[K].ISQAVHAAHAEINEAGTEVVGSAEAGVDAA.[S]		23	0.00259	3	958.80292	5.34	[324-353]
[R].GGLEPINFQTAADQAR.[E]		20	-0.00061	3	563.28418	5.24	[128-143]
[K].LTEWTSSNVMEER.[K]	1xOxidation [M10]	15	0.00084	2	799.36261	3.86	[265-277]
[A].HAEINEAGTEVVGSAEAGVDAA.[S]		15	0.00042	2	1048.9906	6.25	[332-353]
[M].GITDVFSSSANLSGISSAESLK.[I]		14	0.00064	2	1085.54797	5.9	[302-323]
[R].NVLQPSSVDSQTAM.[V]	1xOxidation [M14]	12	0.00095	2	746.85199	4.71	[160-173]
[S].SVDSQTAMVLVNAIVFK.[G]	1xOxidation [M8]	12	0.00019	2	919.49023	5.53	[166-182]
[N].DVYSFSLASR.[L]		11	-0.00038	2	572.78491	2.8	[96-105]
[N].LSGISSAESLK.[I]		10	0.00025	2	546.30103	3.49	[313-323]
[R].NVLQPSSVDSQTAMVLVNAIVFK.[G]	1xOxidation [M14]	9	0.00133	3	826.11005	5.74	[160-182]
[L].VLLPDEVSGLEQLESIINFEK.[L]		9	0.00011	3	791.4248	4.18	[244-264]
[K].PVQMMYQIGLFR.[V]	2xOxidation [M4; M5]	8	0.00031	2	757.87866	3.49	[208-219]
[M].VLVNAIVFK.[G]		8	-0.00003	2	501.82092	3.45	[174-182]
[K].ILELPFASGTM.[S]	1xOxidation [M11]	8	0.00042	2	597.8078	4.12	[230-240]
[M].MYQIGLFR.[V]	1xOxidation [M1]	7	0.00047	2	522.27124	2.87	[212-219]
[K].YNLTSVLMAM.[G]	2xOxidation [M8; M10]	7	0.00049	2	587.77826	3.99	[292-301]
[H].AEINEAGTEVVGSAEAGVDAA.[S]		7	-0.00088	2	980.45984	6.67	[333-353]
[K].ILELPFASGTMS.[M]	1xOxidation [M11]	7	0.00033	2	641.32373	3.48	[230-241]
[T].KPNDVYSFSLASR.[L]		7	0.0003	3	495.25647	4.05	[93-105]
[M].LVLLPDEVSGLEQLES.[I]		6	0.00079	2	870.96771	3.93	[243-258]
[M].LVLLPDEVSGLEQLESIINFEK.[L]		6	0.00086	3	829.12024	3.96	[243-264]
[S].MLVLLPDEVSGLEQLESIINFEK.[L]	1xOxidation [M1]	6	-0.00002	3	878.13116	4.43	[242-264]
[K].YNLTSVLMAMGITDVF.[S]	2xOxidation [M8; M10]	6	0.00131	2	903.93756	4.86	[292-307]
[M].SMLVLLPDEVSGLEQLESIINFEK.[L]	1xOxidation [M2]	6	0.00011	3	907.14197	5.22	[241-264]
[F].SSSANLSGISSAESLK.[I]		6	0.00123	2	769.39008	4.51	[308-323]
[M].AMGITDVFSSSANLSGISSAESLK.[I]	1xOxidation [M2]	6	0.00052	2	1194.58411	5.48	[300-323]
[K].ILELPFASGTMSM.[L]	2xOxidation [M11; M13]	6	0.0004	2	714.84149	4.36	[230-242]
[K].ILELPFASGTMSML.[V]	2xOxidation [M11; M13]	6	0.00042	2	771.38354	3.95	[230-243]
[S].SSANLSGISSAESLK.[I]		5	0.00089	2	725.87372	4.04	[309-323]
[D].EVSGLEQLESIINFEK.[L]		5	0.00067	2	917.97595	3.14	[249-264]
[L].LPDEVSGLEQLESIINFEK.[L]		5	-0.00005	3	720.70715	4.22	[246-264]
[R].NVLQPSSVDSQTAMVLVN.[A]	1xOxidation [M14]	4	-0.0004	2	959.48254	3.57	[160-177]
[K].PNDVYSFSLASR.[L]		4	0.00023	2	678.33337	3.65	[94-105]
[L].PRMKMEEKYNLTSVLMAMGITDVFSSSANLSGISSAESLK.[I]	1xOxidation [M]	4	0.01019	3	1447.05933	3.74	[284-323]
[DY].QIGLFR.[LV]		4	-0.00012	2	367.22128	2.04	[214-219]
[M].LVLLPDEVSGLEQL.[E]		4	0.00001	2	762.92963	3.54	[243-256]
[S].MLVLLPDEVSGLEQL.[E]	1xOxidation [M1]	4	0.00007	2	836.44739	2.54	[242-256]
[K].YNLTSVLMAMGITDVFS.[S]	2xOxidation [M8; M10]	4	0.00073	2	947.453	3.66	[292-308]
[K].YNLTSVLMAMGITDVFSSSAN.[L]	2xOxidation [M8; M10]	4	-0.00064	2	1127.02368	6.96	[292-312]
[K].YNLTSVLMAMGITDVFSSSANLSGISSAESLK.[I]	2xOxidation [M8; M10]	4	0.00015	3	1109.21094	5.44	[292-323]
[R].GGLEPINFQT.[A]		4	-0.00058	2	538.27399	2.95	[128-137]
[M].GITDVFSSSAN.[L]		4	0.00023	2	549.25934	3.55	[302-312]
[H].AAHAEINEAGTEVVGSAE.[A]		4	0.00036	2	877.91357	3.05	[330-347]

[R].DILNQITKPNDVYSF.[S]		4	0.00023	2	883.95184	4.26	[86-100]

Table S2. WT OVA peptides identified by mass spectrometry after trypsin digestion of fragment A (top) fragment B (middle) and fragment C (bottom) proteolytic fragments identified in Fig. 4. Tryptic peptides that overlap with OVA 323-339 are highlighted.

Fragment A

Annotated Sequence	Modifications	# PSMs	Delta m/z [Da]	Charge	m/z [Da]	XCorr	Positions
[K].DEDTQAMPFR.[V]		2	0.00017	2	605.264	2.14	[191-200]
[R].VTEQESKPVQMMYQIGLFR.[V]	2xOxidation [M11; M12]	21	0.00083	3	772.7178	4.92	[201-219]
[R].VTEQESKPVQMMYQIGLFR.[V]	1xOxidation [M]	6	0.00026	3	767.3856	6.17	[201-219]
[R].VTEQESKPVQMMYQIGLFR.[V]		1	-0.00551	3	762.0482	2.59	[201-219]
[R].NVLQPSSVDSQTAMVLVNAIVFK.[G]	1xOxidation [M14]	35	0.00024	2	1238.66	4.7	[160-182]
[R].NVLQPSSVDSQTAMVLVNAIVFK.[G]		3	0.00075	2	1230.663	2.77	[160-182]
[K].LTEWTSSNVMEER.[K]	1xOxidation [M10]	13	0.00047	2	799.3622	3.65	[265-277]
[K].YNLTSVLMAMGITDVFSSSANLSGISSAESLK.[I]	2xOxidation [M8; M10]	1	-0.00021	3	1109.211	3.16	[292-323]
[K].LTEWTSSNVMEER.[K]		3	0.00062	2	791.3649	3.23	[265-277]
[K].HIATNAVLFFGR.[C]		3	-0.00011	3	449.2506	3.31	[371-382]
[R].GGLEPINFQTAADQAR.[E]		108	0.00024	3	563.285	5.07	[128-143]
[R].EVVGSAEAGVDAASVSEEFR.[A]		5	0.0008	3	670.3209	6.07	[341-360]
[K].ELYRGGLEPINFQTAADQAR.[E]		1	0.00038	3	750.3822	5.49	[124-143]
[R].ELINSWVESQTNGIIR.[N]		8	0.00073	2	929.9872	5.92	[144-159]
[R].DILNQITKPNDVYSFSLASR.[L]		5	-0.00018	3	761.0654	5.73	[86-105]
[K].ISQAVHAAHAEINEAGR.[E]		18	0.00009	4	444.2303	5.43	[324-340]
[R].YPILPEYLQCVK.[E]		3	0.00072	2	733.3923	2.42	[112-123]

Fragment B

Annotated Sequence	Modifications	# PSMs	Delta m/z [Da]	Charge	m/z [Da]	XCorr	Positions
[R].DILNQITKPNDVYSFSLASR.[L]		2	0.00049	3	761.0661	3.23	[86-105]
[R].ELINSWVESQTNGIIR.[N]		6	0.00049	2	929.987	4.56	[144-159]
[R].EVVGSAEAGVDAASVSEEFR.[A]		7	0.00068	3	670.3208	6.13	[341-360]
[R].GGLEPINFQTAADQAR.[E]		19	0.0003	3	563.2851	4.71	[128-143]
[K].ILELPFASGTMSMLVLLPDEVSGLEQLESIINFEK.[L]	2xOxidation [M11; M13]	1	-0.00092	3	1299.004	2.46	[230-264]
[K].ISQAVHAAHAEINEAGR.[E]		8	0.00018	4	444.2304	6.02	[324-340]
[K].LTEWTSSNVMEER.[K]	1xOxidation [M10]	6	0.00108	2	799.3629	3.26	[265-277]
[R].NVLQPSSVDSQTAMVLVNAIVFK.[G]		1	0.00094	3	820.778	2.32	[160-182]
[R].NVLQPSSVDSQTAMVLVNAIVFK.[G]	1xOxidation [M14]	16	0.00176	3	826.1105	5.4	[160-182]
[R].VTEQESKPVQMMYQIGLFR.[V]	2xOxidation [M11; M12]	6	0.00035	3	772.7173	5.24	[201-219]
[K].YNLTSVLMAMGITDVFSSSANLSGISSAESLK.[I]	2xOxidation [M8; M10]	11	0.00125	3	1109.212	6.91	[292-323]
[R].YPILPEYLQCVK.[E]		1	0.00066	2	733.3923	2.39	[112-123]

Fragment C

Annotated Sequence	Modifications	# PSMs	Delta m/z [Da]	Charge	m/z [Da]	XCorr	Positions
[R].ELINSWVESQTNGIIR.[N]		1	0.00042	2	929.9869	2.37	[144-159]
[R].EVVGSAEAGVDAASVSEEFR.[A]		8	0.00044	3	670.3206	6.13	[341-360]

[R].GGLEPINFQTAADQAR.[E]		19	-0.00019	3	563.2846	4.8	[128-143]
[K].HIATNAVLFFGR.[C]		2	0.00004	3	449.2507	3.29	[371-382]
[K].ISQAVHAAHAEINEAGR.[E]		17	0.00088	4	444.2311	5.93	[324-340]
[K].LTEWTSSNVMEER.[K]	1xOxidation [M10]	7	0.00066	2	799.3624	3.21	[265-277]
[R].NVLQPSSVDSQTAMVLVNAIVFK.[G]	1xOxidation [M14]	3	0.00078	3	826.1095	3.87	[160-182]
[R].VTEQESKPVQMMYQIGLFR.[V]	2xOxidation [M11; M12]	4	0.00065	3	772.7176	4.86	[201-219]
[K].YNLTSVLMAMGITDVFSSSANLSGISSAESLK.[I]	2xOxidation [M8; M10]	2	0.00003	3	1109.211	7.22	[292-323]
[R].ELINSWVESQTNGIIR.[N]		1	0.00042	2	929.9869	2.37	[144-159]
[R].EVVGSAEAGVDAASVSEEFR.[A]		8	0.00044	3	670.3206	6.13	[341-360]
[R].GGLEPINFQTAADQAR.[E]		19	-0.00019	3	563.2846	4.8	[128-143]
